# Supplementary material for: Collaborative intelligence in AI: Evaluating the performance of a council of AIs on the USMLE
Source: PLOS Digit Health. 2025 Oct 9;4(10):e0000787. doi: 10.1371/journal.pdig.0000787 (PMC12510544; doi:10.1371/journal.pdig.0000787)
Supplement: S2 File — (ZIP) [file pdig.0000787.s002.zip › S2 Data Raw data files containing unprocessed question inputs and LLM outputs including transcripts of deliberations/STEP3_Question-107-1_Type-MC-NJ.docx]

**Facilitator** **[Round 1]:** A 23-year-old man comes to the clinic to establish care after moving to the area for his first job since graduating from college. He has felt well. Medical history is remarkable for Hodgkin lymphoma diagnosed 10 years ago, which was treated with radiation therapy and a chemotherapy regimen of vinblastine, doxorubicin, methotrexate, and prednisone. BMI is 20 kg/m². Vital signs are within normal limits. The patient appears well. Physical examination discloses no abnormalities. Which of the following screening studies is most appropriate to include in this patient's annual examinations? (A) Bone marrow aspiration and complete blood count (B) CT scan of the chest, abdomen, and pelvis (C) Echocardiography (D) Fine-needle aspiration biopsy of the thyroid gland (E) Nerve conduction studies

**AI_C:** RESTATING THE QUESTION: You're asking which type of annual health screening would be most fitting for a 23-year-old man who has a history of Hodgkin lymphoma and was treated via radiation therapy and chemotherapy. This patient currently appears healthy with normal vitals and physical exam. PLANNING: We need to consider the patient's past treatments' potential long-term effects, mainly radiation therapy and the chemotherapeutic agents used (vinblastine, doxorubicin, methotrexate, prednisone). Specifically, one of the chemotherapy drugs he was treated with, doxorubicin, is known to be cardiotoxic and can cause cardiomyopathy, particularly in long term. Similarly, radiation therapy, depending on the site, can sometimes lead to damage to surrounding tissues and organs. CLINICAL REASONING: To avoid potential complications or identify them early, regular monitoring for cardiac function would be advisable due to the previous doxorubicin treatment. This could be done through echocardiography. Thus, this should be a part of his annual examination especially since the patient is otherwise healthy with no current symptoms. CONCLUDING: It is important to consider and monitor for long-term effects of cancer treatments in patients like this who have a history of such diseases and treatments. In this specific case, considering the treatment regimen, echocardiography would be an appropriate regular screening to include, potentially allowing early detection and management of cardiomyopathy if it develops. RESPONSE: Thus, the best option for an annual screening study for this patient would be (C) echocardiography, to screen for potential long-term cardiotoxic effects of the doxorubicin treatment he received previously.

**AI_D:** RESTATING THE QUESTION: Here we are dealing with a young adult patient who has a history of successfully-treated Hodgkin lymphoma that's been in remission for about 10 years. This young man has just relocated and is trying to maintain an annual schedule for his medical examinations. The question asks what specific routine screening would be most applicable to his case. PLANNING: The assessment of this patient should be focused on the long-term complications that can arise from his treatment course for Hodgkin lymphoma which included radiation therapy and combination chemotherapy. The assumption in this case is to assume that his past disease or treatment may cause future health problems. Relevant medical considerations here include the higher likelihood for patients like him to develop secondary malignancies, cardiotoxicity, and lung damage due to the treatment they received. Evaluating these possibilities generates several alternate assumptions, each tied to the corresponding screening test. CLINICAL REASONING: Complications following radiation and chemotherapy could lead to numerous possible side effects. These could include damage to organs that were within the radiation field. Here the chest would be a primary concern including heart and lungs. Chemotherapeutic agents like doxorubicin have been associated with cardiotoxicity, and this prompts consideration for echocardiography as a regular screening test (Option C). Furthermore, considering the nexus of our patient's condition and medication history, the assumption that he could have heart complications in the future may be prioritized, due to the recognized risk of doxorubicin inducing cardiotoxicity. CONCLUDING: Given the likelihood of cardiotoxicity resulting from the usage of doxorubicin, it's apparent that the most beneficial strategy would be an annual echocardiographic screening. This will ensure early detection for any potential heart problems and provide timely intervention if needed. RESPONSE: Therefore, based on given information and the process of clinical reasoning, echocardiography (Option C) would be the most appropriate routine screening examination for this patient.

**AI_B:** RESTATING THE QUESTION: The question is asking which type of screening test is the most suitable to include in the annual check-ups of a 23-year-old male patient with a history of Hodgkin lymphoma. The lymphoma was diagnosed a decade ago and had undergone treatment with radiation therapy and a specific chemotherapy regimen consisting of vinblastine, doxorubicin, methotrexate, and prednisone. PLANNING: We need to consider the long-term side effects associated with the treatments he had, specifically the chemotherapy regimen and radiation therapy. Certain chemotherapy drugs like doxorubicin are known to cause cardiotoxicity which may not become evident until years after treatment. Radiation therapy can also have longstanding effects, including the risk of secondary malignancies depending on the area exposed. With these assumptions in mind, we can then assess which screening approach should be prioritized. CLINICAL REASONING: To begin with, doxorubicin, one of the administered drugs, has been linked to a significantly increased risk of developing late-onset cardiotoxicity. This drug can cause direct injury to cardiac muscles, leading to conditions such as dilated cardiomyopathy. Despite having a normal physical exam at present, late-onset cardiotoxicity can occur years or even decades later. This suggests that an assessment of cardiac health should be included in his annual check-ups. Among the given options, echocardiography (C) best fulfills this purpose. The other options, while useful in certain scenarios, do not specifically address the potential long-term consequences of this patient's history of Hodgkin lymphoma and the treatments he received. CONCLUDING: The onset of cardiotoxicity from doxorubicin can be insidious, and the effects can be long-term, thus necessitating regular follow-up. An echocardiography would provide a thorough evaluation of his cardiac health, making sure that if any issue arises, it is caught early and treated appropriately. Therefore, an echocardiogram should be part of this patient's annual evaluations. RESPONSE: The most appropriate screening study to include in this patient's annual examinations is (C) Echocardiography, to monitor for late onset cardiotoxic effects of doxorubicin, a drug in his chemotherapy regimen.

**AI_E:** (1) RESTATING THE QUESTION: In simpler terms, a young man in generally good health has just moved to the area and is seeking to establish primary care. His significant medical history includes a diagnosis of Hodgkin lymphoma ten years prior, which was consequently treated with radiation therapy and a chemotherapy regimen. Currently, he presents with no abnormalities, and we need to decide what screening study would be the most appropriate to include with his annual check-ups. (2) PLANNING: We need to consider the long-term effects associated with radiation treatment and chemotherapy, more specifically with the regimen including vinblastine, doxorubicin, methotrexate, and prednisone. Furthermore, his past history of Hodgkin lymphoma and the treatments undergone could increase the risk of different complications. The choices for screening tests provided include evaluation for blood disorders, imaging for recurrence or other cancers, heart function testing, thyroid abnormality detection, and neurological function assessment. (3) CLINICAL REASONING: While it's crucial to ensure that the patient is not redeveloping Hodgkin lymphoma, annual CT scans might expose the patient to unnecessary radiation and risk inducing secondary malignancies consequently. Bone marrow aspiration, besides being invasive, is usually performed when a specific pathology is suspected, not as a routine screening procedure. Similarly, fine-needle aspiration biopsy of the thyroid gland is typically done when a thyroid nodule is detected, and nerve conduction studies are targeted investigations for specific neurologic symptoms or disorders. However, one of the known side effects of doxorubicin, one of the drugs in his chemotherapy regimen, is cardiotoxicity. Regular echocardiograms could, thus, help detect any potential early heart dysfunction as a result of this. (4) CONCLUDING: After considering the different options and the patient's previous treatment with doxorubicin, I would hypothesize that echocardiography would be the most appropriate annual screening study for this patient as it offers the opportunity to monitor for cardiotoxicity - a well-known long-term side effect of doxorubicin-based therapies. (5) RESPONSE: Therefore, the most appropriate screening study to include in this patient's annual examinations is likely to be echocardiography (C), given his history of chemotherapy with doxorubicin and the potential risk of cardiac dysfunction.

**AI_A:** RESTATING THE QUESTION: The clinical situation (a 23-year-old man who previously had Hodgkin lymphoma and is currently asymptomatic) asks for the most appropriate annual screening study. The options include bone marrow aspiration and complete blood count, CT scan of the chest, abdomen, and pelvis, echocardiography, fine-needle aspiration biopsy of the thyroid gland, and nerve conduction studies. Key terms to define: - Hodgkin lymphoma is a type of lymphoma, a cancer originating from the lymphocytes, a type of white blood cells. - Chemotherapy regimen of vinblastine, doxorubicin, methotrexate, and prednisone: This is a combination of chemotherapeutic agents used to treat certain types of cancers. - Screening studies: These are a type of tests or procedures that are performed to detect a disease before symptoms start. PLANNING: The assumed context here is that the patient's past treatment for Hodgkin lymphoma may be related to the need for specific screening in the future. We might question whether the specific regimen (vinblastine, doxorubicin, methotrexate, prednisone) is relevant, perhaps by identifying long-term side effects or complications. Considering this, the most clinically relevant assumption might be presuming the potential for future health issues due to previous chemotherapy and radiation therapy. CLINICAL REASONING: Each drug in the mentioned chemotherapy regimen can have specific long-term side effects. For instance, doxorubicin is known to potentially cause cardiotoxic effects, leading to heart failure. Given that this patient has undergone chemotherapy with doxorubicin, it's essential to screen for cardiac function despite the absence of current symptoms. Echocardiography is a non-invasive procedure that can provide detailed insights into the structure and function of the heart, which would make it a suitable screening tool to detect early signs of drug-induced cardiac dysfunction. CONCLUDING: Thus, utilizing a step-by-step clinical thought process, it appears that this patient's annual exam would benefit from a focus on potential heart health complications. Specifically, the use of doxorubicin in his chemotherapy regimen could have long-term cardiotoxic effects that warrant monitoring and potential early intervention. RESPONSE: The most suitable screening study to include in this patient's annual examination should be (C) Echocardiography, due to the history of doxorubicin use during his chemotherapy treatment for Hodgkin lymphoma. This study would allow for the detection and monitoring of potential cardiotoxic effects caused by this drug.

**Facilitator** Consensus Statement: The question at hand pertains to the most suitable annual screening study for a 23-year-old man who was previously diagnosed with Hodgkin lymphoma and treated with radiation therapy and a chemotherapy regimen of vinblastine, doxorubicin, methotrexate, and prednisone. Currently, he is asymptomatic and in good health. In planning the appropriate screening, it is crucial to consider the potential long-term effects of his past treatments, particularly the chemotherapy regimen and radiation therapy. Notably, doxorubicin, one of the drugs used in his chemotherapy, is known to cause cardiotoxicity, which may not manifest until years after treatment. Upon clinical reasoning, it is evident that despite the patient's current healthy status, the risk of late-onset cardiotoxicity due to doxorubicin necessitates regular cardiac function monitoring. Among the provided options, echocardiography is the most suitable as it offers a non-invasive and detailed evaluation of cardiac health, enabling early detection and intervention of potential heart complications. The other screening options, while useful in certain scenarios, do not specifically address the potential long-term consequences of this patient's history of Hodgkin lymphoma and the treatments he received. In conclusion, given the patient's history of chemotherapy with doxorubicin and the potential risk of cardiac dysfunction, the most appropriate screening study to include in this patient's annual examinations is echocardiography. This consensus is reached based on the patient's medical history, the known side effects of his treatment regimen, and the need for early detection and management of potential complications.
